# Acute Necrotizing Pancreatitis Presenting as Gastrocolic Fistula: A Rare Occurrence

**DOI:** 10.7759/cureus.53937

**Published:** 2024-02-09

**Authors:** Twinkle Pawar, Harshitha Reddy, Sunil Kumar, Sourya Acharya, Vijendra Kirnake

**Affiliations:** 1 Department of Medicine, Jawaharlal Nehru Medical College, Datta Meghe Institute of Medical Sciences (Deemed to be University), Wardha, IND; 2 Department of Gastroenterology, Jawaharlal Nehru Medical College, Datta Meghe Institute of Medical Sciences (Deemed to be University), Wardha, IND

**Keywords:** case report, gastrocolic fistula, hematochezia, necrosis, pancreatitis

## Abstract

Tissue necrosis and ischemia are hallmarks of acute necrotizing pancreatitis, which frequently results in fatal infections. In this case, we describe a man in his 40s who had diffuse pain in the abdomen, intractable vomiting, diarrhoea, and intermittent fever. His abdominal computed tomography revealed acute pancreatitis with peripancreatic fluid collection, gastric perforation, and fistula formation between the greater curvature of the stomach and transverse colon. His upper gastrointestinal (GI) endoscopy confirmed a gastrocolic fistula.

## Introduction

The symptoms of acute pancreatitis include nausea, vomiting, and pain in the epigastrium that frequently radiates to the back. Etiology for pancreatitis are gallstones, alcohol, idiopathic, autoimmune, and drug-induced, bacterial infections like *Salmonella typhi*, viral infections like varicella, and fungal infections [1-3]. An extreme form of acute pancreatitis brought on by necrosis and tissue ischemia is known as acute necrotizing pancreatitis (ANP). It frequently results in high mortality and dangerous infections [4]. ANP's consequences include bleeding, compressive symptoms in the duodenum, colon, and portal vein, and the collection spreading throughout the peritoneal or retroperitoneal cavity [5]. There are two natural stages to the progression of severe acute pancreatitis. The first two weeks are marked by systemic inflammatory response syndrome due to the generation of inflammatory mediators. In patients with ANP, organ failure is prevalent and often occurs without infection [6]. The second stage of the illness, which starts about two weeks following the first, is characterized by sepsis-related problems brought on by infection-induced pancreatic necrosis. Intervention is usually required for patients with sterile necrosis who are symptomatic and infected with pancreatic necrosis [7].

## Case presentation

A 44-year-old male was brought to the casualty with complaints of diffuse abdominal pain, intractable vomiting following a meal, diarrhoea, and intermittent fever for 12 days. The patient also complained of generalized weakness and weight loss. He was a chronic alcoholic for 15-20 years.

The patient denied any history of hypertension, diabetes mellitus, bronchial asthma, tuberculosis, or acquired immunodeficiency syndrome. He had previously complained about similar symptoms four to five years ago.

On general examination, the patient was febrile and had mild icterus; pulse was 124/min, and blood pressure was 100/70 mmHg.

On systemic examination of the patient's upper abdomen, there were tenderness, a local rise of temperature, and guarding. His bowel sounds were absent. The cardiovascular system was normal, apart from tachycardia. The respiratory system revealed absent breath sounds bilaterally in all the lung fields. The central nervous system was normal.

All the basic laboratory parameters of the patient on admission are shown in Table 1.

**Table 1:** All the basic laboratory parameters of the patient on the day of admission.

Laboratory investigations	Value in the patient	Biological reference range
Hemoglobin	6.8	13-15 g/dL
Total leucocyte count	29,800	4000-11000/cumm
Platelet count	3,89,000	1,50,000-4,50,000/cumm
Mean corpuscular volume	89.3	79-100 fL
Serum urea	24	9-20 mg/dL
Serum creatinine	0.8	0.6-1.2 mg/dL
Serum sodium	128	135-145 mmol/L
Serum potassium	3.6	3.5-5.1 mmol/L
Serum alkaline phosphatase	424	38-126 U/L
Serum alanine transaminase	830	<50 U/L
Serum aspartate transaminase	690	17-59 U/L
Serum total protein	3.6	6.3-8.2 g/dL
Serum albumin	2.2	3.5-5 g/dL
Serum total bilirubin	5.4	0.2-1.3 mg/dL
Serum globulin	1.4	2.3-3.5 g/dL
Activated partial thromboplastin clotting time	68.1	29.5 control
Prothrombin time	20	11.9 control
International normalized ratio	1.56	0.8-1.2
Serum ammonia	104	9-30 µmol/L
Highly sensitive C-reactive protein	9	1-3 mg/dL
Lactate dehydrogenase	639	120-246 U/L
Serum amylase	476	30-110 U/L
Serum lipase	789	23-300 U/L

His ultrasonography of the abdomen revealed a bulky pancreas showing altered echotexture with >30% of the necrotic area in the body and tail region and moderate peripancreatic inflammation suggestive of ANP, with cholelithiasis and diffuse altered echotexture of the liver.

**Figure 1:**
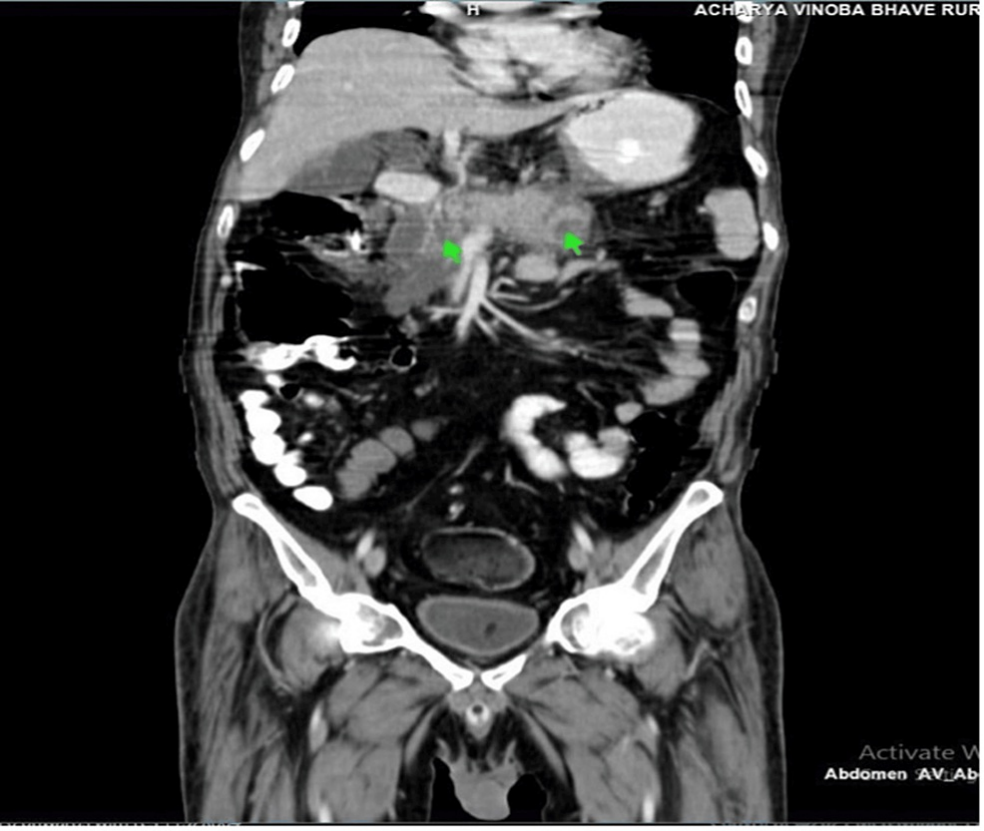
The pancreas is swollen, and there are non-enhancing areas in the neck and body of the pancreas suggestive of acute necrotizing pancreatitis (green arrows).

His contrast-enhanced computed tomography (CT) of the abdomen done on the day of admission revealed acute pancreatitis with areas of necrosis within the pancreas and peripancreatic fluid, fat stranding, pseudo-pancreatic cyst, small pockets of air in the peritoneal cavity, fatty liver, cholelithiasis, basal lung atelectasis, and pleural effusion bilaterally. There was significant thinning of the anterior and inferior walls of the stomach associated with concealed collection adjacent to the omentum as shown in Figure 1.

**Figure 2:**
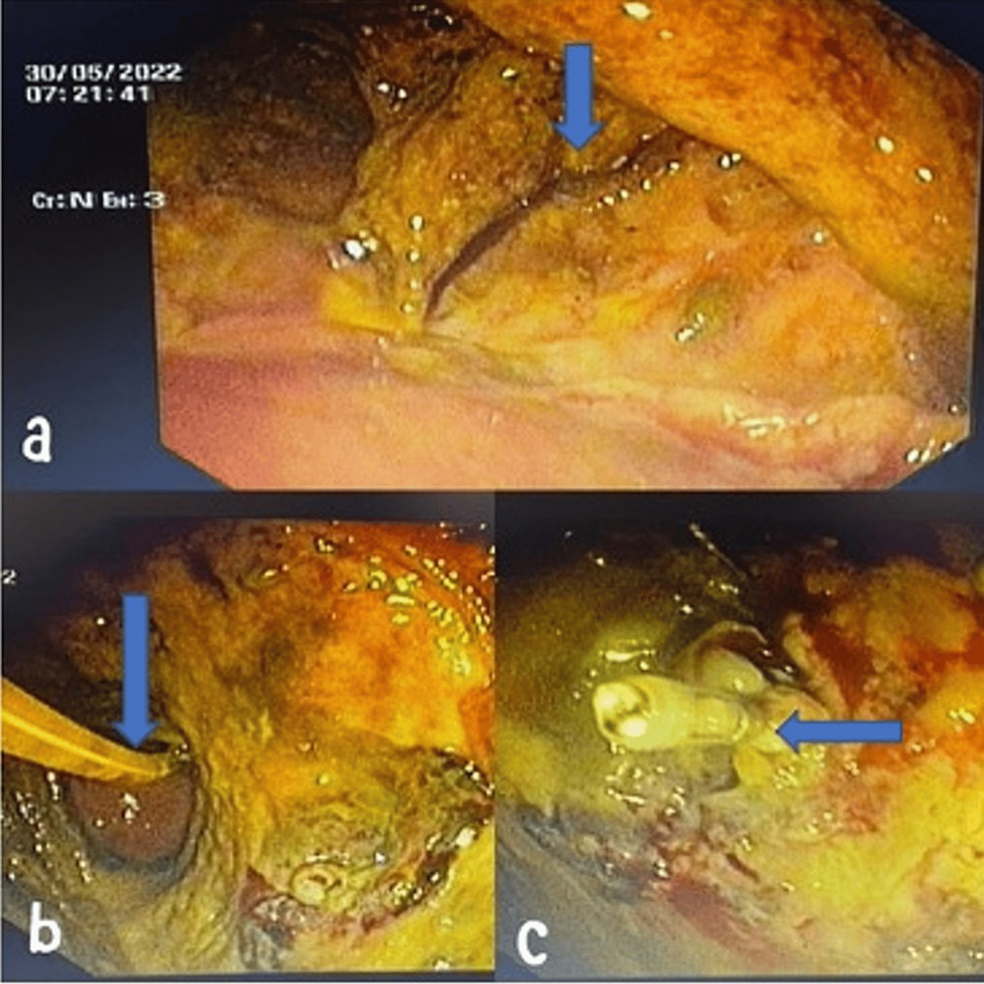
(a) Ulceration in the body and antrum of the stomach. (b) The nasojejunal tube could be seen in communication with the stomach, small bowel (likely the jejunum), and colon. (c) Hemoclip application.

In view of the patient's clinical deterioration and new complaints of hematemesis and hematochezia, an upper gastrointestinal (GI) endoscopy was done, which revealed a diffuse ulcer in the body and antrum of the stomach (Figure 2a).

The patient then had a fall in hemoglobin and platelet counts, and a surgical consult was taken for necrotizing pancreatitis.

In view of hematemesis and hematochezia, angiography was done, which was suggestive of a small aneurysm arising from the left gastric artery. An interventional radiologist's opinion was taken, and embolization of the gastroduodenal artery aneurysm was done.

Even after embolization, the patient still had persistent episodes of hematemesis and hematochezia.

Nasojejunal tube placement was done, and sigmoidoscopy was done. The stomach, small bowel (perhaps the jejunum), and colon were all connected to the necrotic cavity due to a fistula. The nasojejunal tube can be seen in Figure 2b. There was a visible vessel with a blood clot around it. 20cc of adrenalin was injected around the vessel into the necrotic area. Hemoclip was applied over the vessel's base, as shown in Figure 2c.

In spite of the hemoclip insertion, the patient still complained of hematochezia, and his clinical condition deteriorated. He was advised exploratory laparotomy but was deferred due to financial and family constraints and was discharged against medical advice. Eventually, the patient was lost to follow-up. 

## Discussion

Because necrosis is linked to organ failure and infectious complications, it raises the risk of morbidity and mortality in acute pancreatitis. Fistula development is one of the frequently described complications of severe ANP. The two organs where fistulas most frequently occur are the colon and duodenum [5]. Communication typically occurs in a gastrocolic fistula (GCF) between the greater curvature of the stomach and the distal transverse colon. Gastric and colon cancers are the most frequent causes of GCF. Additionally, it can happen in cases of chronic pancreatitis, peptic ulcer disease, inflammatory bowel disease, diverticular disease, and pancreatic abscess [6,7].

Although severe acute pancreatitis-related GCF is not frequently documented, it can develop as a result of surgery, direct erosion by pancreatic enzymes, or intestinal necrosis brought on by vascular thrombosis. Malnutrition, infection, and GI bleeding are complications of GCF. However, the presence of a GCF can complicate the clinical picture, causing unusual symptoms like persistent diarrhoea, feculent vomiting, weight loss, and electrolyte imbalances due to altered GI transit. Confirming the presence of a GCF can be challenging. Various imaging modalities, including CT scans with contrast enhancement, upper GI series, and endoscopic procedures like endoscopic retrograde cholangiopancreatography (ERCP) or endoscopic ultrasound, are often employed to visualize the abnormal connection between the stomach and colon and assess the extent of pancreatic necrosis [7,8].

Initial steps involve aggressive fluid resuscitation, pain management, nutritional support, and the administration of antibiotics to prevent infections related to pancreatic necrosis, and surgery is still the go-to therapy [8]. The fistulous tract is typically resected with a wedge-shaped segment of the stomach and a portion of the colon. For patients who are severely disabled, a diversion colostomy with fistulous tract excision is reserved [9,10]. 

## Conclusions

One deadly side effect of ANP is a GCF. In this case report, we highlighted the late diagnosis of ANP, which has led to GCF, thence the poor prognosis of the patient. This case report will increase awareness among general physicians, and its early recognition and management may help increase the patient's chances of survival.
